# International physician survey on management of FOP: a modified Delphi study

**DOI:** 10.1186/s13023-017-0659-4

**Published:** 2017-06-12

**Authors:** Maja Di Rocco, Genevieve Baujat, Marta Bertamino, Matthew Brown, Carmen L. De Cunto, Patricia L. R. Delai, Elisabeth M. W. Eekhoff, Nobuhiko Haga, Edward Hsiao, Richard Keen, Rolf Morhart, Robert J. Pignolo, Frederick S. Kaplan

**Affiliations:** 1 Department of Pediatrics, Unit of Rare Diseases, Giannina Gaslini Institute, Largo Gaslini 5, 16147 Genoa, Italy; 2 Service of Medical Genetics CHU Paris - Hôpital Necker-Enfants Malades, 149 rue de Sèvres, 75743 Paris, France; 3 Institute of Health and Biomedical Innovation, Queensland University of Technology, Translational Research Institute, Princess Alexandra Hospital, Woolloongabba, QLD 4069 Australia; 4 Department of Pediatrics, Pediatric Rheumatology Section, Hospital Italiano de Buenos Aires, Gascón 450, 1181 Ciudad Autónoma de Buenos Aires, Argentina; 5 Orthopaedic Department of Santa Casa de Misericórdia de São Paulo, School of Medicine Faculdade de Ciências Médicas da Santa Casa de São Paulo, Rua Pedro de Toledo 129 cj 121, Vila Clementino, 04039-001 São Paulo Brazil; 6 Department of Internal Medicine/Section Endocrinology, VU Medical Center Amsterdam, De Boelelaan 1117, 1081 HV Amsterdam, The Netherlands; 7 Department of Rehabilitation Medicine Graduate School of Medicine, The University of Tokyo, 7-3-1 Hongo, Bunkyo-ku, Tokyo, 113-8655 Japan; 8 Department of Endocrinology, Faculty Practice University of California-San Francisco, 400 Parnassus Ave., San Francisco, CA 94143-1222 USA; 9 University College London Hospitals, London, NW1 2PQ UK; 10 Department of Pediatrics Klinikum Garmisch-Partenkirchen GmbH, Auenstraße 6, 82467 Garmisch-Partenkirchen, Germany; 11 Department of Medicine, Division of Geriatric Medicine & Gerontology, Mayo Clinic College of Medicine, 200 First Street SW, Rochester, MN USA; 12 Department of Orthopaedic Surgery, Center for Research in FOP & Related Disorders, The Perelman School of Medicine, The University of Pennsylvania, 3737 Market Street, Philadelphia, PA 19104 USA

**Keywords:** Fibrodysplasia ossificans progressiva, ACVR1

## Abstract

Fibrodysplasia ossificans progressiva (FOP), a disabling disorder of progressive heterotopic ossification (HEO), is caused by heterozygous gain-of- function mutations in Activin receptor A, type I (ACVR1, also known as ALK2), a bone morphogenetic protein (BMP) type I receptor. Presently, symptomatic management is possible, but no definitive treatments are available. Although extensive guidelines for symptomatic management are widely used, regional preferences exist. In order to understand if there was worldwide consensus among clinicians treating FOP patients, an expert panel of physicians directly involved in FOP patient care was convened. Using a modified Delphi method, broad international consensus was reached on four main topics: diagnosis, prevention of flare-ups, patient and family-centered care and general clinical management issues. This study of physician preferences provides a basis for standardization of clinical management for FOP.

## Background

Fibrodysplasia ossificans progressiva (FOP; MIM #135100) is a rare and disabling genetic condition of progressive heterotopic ossification with a point prevalence of approximately 1 per 2 million. No ethnic, racial, gender or geographic predisposition has been reported [1, 2]. FOP results from mutations in the intracellular domain of the bone morphogenetic protein (BMP) type I receptor, ACVR1, which drive extraskeletal bone formation [3–5]. Mutant (mt) ACVR1 exhibits mild basal hyperactivity and hyper-responsiveness to canonical and non-canonical ligands [6–8].

Classic FOP, seen in ~97% of patients worldwide is characterized by two clinical features: malformations of the great toes and progressive heterotopic endochondral ossification (HEO), and by a single missense activating mutation in ACVR1 (c.617G > A;R206H) [1–4]. Misdiagnosis is common [9]. Clinical and genetic variants have been described [2, 4]. Malformations of the great toe are noted at birth; neck stiffness due to orthotopic fusion of cervical vertebra is a common early finding. Other skeletal anomalies associated with FOP include short malformed thumbs, clinodactyly, short broad femoral necks, hip dysplasia and proximal medial tibial osteochondromas [4].

Most individuals with classic FOP experience episodes of painful inflammation of soft tissues (flare-up), followed by heterotopic ossification of skeletal muscle, fascia, tendons, aponeuroses, and ligaments beginning in the first decade of life. Progressive joint dysfunction without flare-up can also occur, with or without HEO. Extraskeletal bone formation is permanent and leads to progressive immobility and need for assistance in performing activities of daily living [10]. Finally, progressive restrictive chest wall disease and secondary cardiac failure lead to death, commonly in the fifth decade [11]. At present, no definitive treatment is available; symptomatic management prevails but varies widely [12]. Basic and clinical research is identifying robust targets for clinical development [1, 7, 8, 13–16].

In order to better understand if there is worldwide consensus among clinicians in FOP patient care, an expert panel of physicians directly involved in patient management was convened at an international FOP patient meeting to address this issue.

## Main text

The consensus process incorporated a five-step modified Delphi method, which took place between January and June 2016 [17]. First, a thorough review of the literature and FOP Treatment Guidelines was performed by the principal investigator (PI; MDR). Second, an e-mail questionnaire, with a comprehensive list of statements related to four main topics (diagnosis, prevention of flare-ups, patient and family-centered care and general clinical management issues) was established by the PI. Third, a panel of ten international experts was convened by the PI based on physician participation at the annual FOP patient and family meeting of FOP Italia in Livorno, Italy in April, 2016. The panel included physicians from ten countries (Argentina, Australia, Brazil, France, Germany, Italy, Japan, Netherlands, United Kingdom and the United States) and from five continents (North America, South America, Europe, Asia and Australia). Fourth, the expert panel was asked to rank, revise and order statements for consideration. Finally, the panel was asked to indicate agreement, partial agreement or disagreement with specific statements agreed upon for assessment.

Twenty statements considered by the expert panel received at least consensus approval and are reported in Table 1.

**Table 1:** Consensus statements

*Diagnosis*
Definitive diagnosis of FOP requires genetic confirmation Infants or children diagnosed before the onset of flare-ups need annual clinical assessment In FOP patients diagnosed before the onset of flare-ups, flare-up prevention is recommended
*Prevention of flare-ups*
The prevention of flare-ups involves recognizing known causes of flare-up (blunt muscle trauma, muscle fatigue, muscular stretching, intramuscular injections) In case of blunt muscle trauma, oral prednisone, for at least 3 days, should be considered to prevent flare-up Steroid prophylaxis is recommended for dental and surgical procedures There is no evidence that chronic treatment with NSAIDs prevents flare-ups or HEO Immunization by subcutaneous administration is recommended for all vaccines which can be administered by that route. For the other vaccines, risk/benefit of intramuscular administration should be discussed with patients or parents. No immunizations should be given during flare-ups In dental procedures, overstretching of the temporomandibular joint and mandibular blocks must be avoided Activity is encouraged at all ages, but passive range of motion must be avoided
*Patient & family care*
Each patient should have a primary physician; not necessarily an FOP expert Patients and families should be informed about the IFOPA and country-specific support groups at the time of diagnosis Patients and their families should be educated about safe dental care
*General clinical management issues*
FOP patients should be screened in childhood by audiometry for hearing impairment Occupational therapy, focused on enhancing activities of daily living, may be useful to improve the quality of life of FOP patients In patients with limited motion, prevention of pressure sores by appropriate devices or methods is recommended In patients with respiratory insufficiency, immunization for influenza and pneumococcal pneumonia should be considered An expert anesthesiologist experienced in general anesthesia for FOP patients must be consulted pre-operatively in all cases. Naso-tracheal fiberoptic intubation is the preferable mode of general anesthesia even with intact mouth opening In case of ankylosis of the jaw, a dietician should be consulted to ensure adequate nutrition If clinical examination suggests depression, psychological support is recommended

### Diagnosis

There was general agreement on the need for genetic diagnosis in FOP. However, one expert strongly disagreed and stated “the diagnosis of FOP is clinical.” Other experts agreed conceptually on the need for genetic diagnosis but deferred on practical grounds because genetic analysis was not available in their countries. Most agreed that even if the diagnosis of FOP was clinically certain, genetic confirmation was desirable due to possible phenocopies. All agreed that genetic diagnosis was essential for patients with toe malformation without HEO, in order to exclude those with toe malformations who did not have FOP. All experts agreed that an international effort by the FOP community should ensure genetic testing be available to all individuals who are clinically suspected of having FOP even if they live in countries where no genetic analysis of ACVR1 is available.

### Prevention of flare-ups

All experts agreed that blunt muscle trauma, muscle fatigue, or stretching of the joints or soft tissues should be avoided for fear of triggering flare-ups. Activities with a high risk of injury or falling, such as running, bicycling, or contact sports should also be avoided to the extent possible, but within the developmental norms for a patient’s age group (i.e., children should be allowed to achieve their developmental milestones that involve playing). Individualized lifestyle plans to avoid trauma should be considered for each patient, based on age, limited mobility and cultural norms.

In case of clinically significant impact injuries, early administration of oral prednisone may be useful to prevent flare-ups. Despite lack of stringent clinical evidence, the experts agreed that a brief (3–4 day) course of oral corticosteroids (i.e. prednisone at 2 mg/kg/day), started within the first 24 h after major trauma had a plausible preventive effect [10, 12]. Due to the fact that major dental work or surgery is a high-risk trigger for flare-ups, a course of oral steroids before and after major dental work or surgery could have a positive preventive effect [10, 12].

All experts agreed that intramuscular injections are dangerous for FOP patients because they may trigger a flare-up and heterotopic ossification at the injection site. To prevent flare-ups, subcutaneous immunization for vaccines that can be administrated by the subcutaneous route is advisable; for all the other vaccines, the risk–benefit ratio of intramuscular administration should be discussed with patients and parents or families. All experts agreed that no immunization should be administered during a flare-up [12].

There was general disagreement about chronic treatment with existing medications such as non-steroidal anti-inflammatory drugs to reduce flare-ups. No adequate data exist in FOP animal models or in clinical studies. However, physician discretion in using medications to control disease symptoms in individual patients is advised, as indicated in the FOP Treatment Guidelines [12].

### Patient and family-centered care

The panel of experts agreed with the over-arching goal of patient and family-centered care. Patient and family knowledge, values, beliefs and cultural backgrounds should be incorporated into the planning and delivery of care of FOP patients, as with any patient. The physician should communicate and share complete and unbiased information with patients and families in ways that are affirming and useful and in order to allow the patient to effectively participate in the decision-making process.

As with any rare disease, a multidisciplinary approach to comprehensive medical care and social support by different specialists is advisable. However, each patient should seek and establish a primary physician to coordinate all care. The panel of experts agreed that at least one national or regional center with medical, surgical, anesthesia, physical therapy, occupational therapy and dental expertise in FOP should be identified to optimize care for FOP patients and to minimize risks from any needed medical care and procedures. For continuity of care, knowledge and information concerning the patient specifically and FOP in general should be immediately available to the family doctor as well as to other locally-treating physicians. An emergency card provided to the patient and/or the family is useful in order to summarize FOP-specific issues/concerns.

At the time of diagnosis, patients and families should be informed about the International FOP Association (IFOPA - the primary portal for announcing FOP related clinical trials http://www.ifopa.org) as well as country-specific and language-specific FOP organizations*.* This recommendation is critical for providing patient and family support resources and for facilitating access to a network of patients and providers familiar with FOP.

### General clinical management issues

Annual clinical assessment of infants, children and adults with FOP is preferable but not always achievable. Dental prophylaxis and routine follow-up should begin as early as possible. The education of patients and their families on safe dental care and prophylaxis is essential, especially during childhood years, focusing on hygiene and prevention of caries. Details concerning oral care are available in the FOP Treatment Guidelines [12].

The expert panel reached consensus about major dental procedures (routine extraction of wisdom teeth, root canal, etc.) which should be avoided. Mandibular blocks must be avoided due to the risk of HEO and temporo-mandibular joint ankylosis. Routine dental procedures (cleaning, carrie fillings) should proceed with caution. In case of locked jaw, the consultation of a dietician to ensure adequate nutrition is desirable.

In case of procedures requiring general anesthesia, overstretching of the temporal-mandibular joint must be avoided. Most FOP patients have fusion of cervical vertebrae, limited mouth opening or ankylosis of the temporal-mandibular joint, making oral intubation impossible. In these cases, naso-tracheal fiberoptic intubation is preferred. In patients who retain mouth opening, overstretching of the temporal-mandibular joint can cause flare-ups and ossification; therefore even in these patients awake, naso-tracheal fiberoptic intubation is preferred. An anesthesiologist highly experienced with general anesthesia for FOP patients must be consulted pre-operatively and an experienced otolaryngologist must be available in case tracheostomy is necessary prior to fiberoptic intubation. The positioning of the patient must be meticulous and the use of an air warming blanket or a padded transfer mattress is to be considered in order to minimize soft tissue trauma [12, 18].

Pulmonary function evaluation should begin after 6 years of age, if possible. The Expert Panel does not recommend routine radiographic follow-up due to the unjustified radiation risk-benefit ratio.

Approximately 50% of FOP patients develop hearing impairment during childhood. Audiometry should be considered if clinically warranted. In case of hearing loss that interferes with learning or activities of daily living, the use of hearing aids should be considered [19].

In order to prevent or delay the onset of muscle atrophy secondary to mobility limitations, light physical activity without risk of muscle trauma or stretching (e.g., swimming), that allows patients to perform active range of motion exercise in a safe, low-impact environment, is recommended. Traditional physiotherapy, including passive range of motion, should be avoided, due to the risk of over-stretching and soft tissue injuries. Nevertheless, diaphragmatic breathing exercises and postural re-education can be performed when possible, in order to maintain and improve respiratory function, especially in older patients in whom chest wall dynamics is severely compromised [12].

Occupational therapy, focused on enhancing activities of daily living, may be useful to improve the quality of life of FOP patients. In addition, the goals of physical therapy and any adaptive prosthetics should be clearly focused on patient comfort, rather than trying to correct biomechanical or anatomic defects [12].

In patients with HO, the use of appropriate methods is recommended in order to reduce pressure over bony prominences. In patients with limited motion, frequent position changes are essential for preventing pressure sores. Other strategies include keeping the area clean in order to prevent infections, monitoring skin conditions and maintaining adequate nutrition [12].

The panel reached consensus that the identification of depression, anxiety or other emotional problems, as well as parenting stress and coping behaviors should prompt psychological and/or specialized social service support.

## Conclusion

There was broad consensus on most of the statements considered by the panel (Table 1). However, there were some notable exceptions including – but not limited to – the chronic use of non-steroidal anti-inflammatory medications to prevent HEO. The expert panel agreed to disagree on this contentious issue but reached consensus on the need for additional evidence-based data.

There are many issues regarding the management of FOP that were not addressed in this research study. The exclusion of management issues related to FOP should not be construed as a failure to reach consensus. Many topics were beyond the scope of this ad hoc study.

First, the panel of experts assembled was limited to those attending the FOP Italia Patient and Family Meeting in Livorno, Italy in 2016 and did not necessarily reflect the totality of global expertise in this field. Second, the generation of topics for consideration focused primarily on prevention and purposely circumvented controversial topics of symptomatic management such as palliative medications. Third, the FOP Treatment Guidelines, a consensus document published by clinical experts worldwide, and whose revision is anticipated in 2018, will comprehensibly address the central management issues in FOP as it has in the past [12]. Nevertheless, this survey will assist physicians in the contemporary clinical management of FOP patients, circumscribe critical areas of preventive management, and inform future consensus guidelines. Finally, the consensus statements reported in this study are not intended to substitute for the independent professional judgment of treating physicians as they cannot account for regional heterogeneity of access to clinical care or the best advice and guidance for individual FOP patients and families.

## Abbreviations

FOP: Fibrodysplasia ossificans progressive
HEO: Heterotopic ossification
